# ASAP ECMO: Antibiotic, Sedative and Analgesic Pharmacokinetics during Extracorporeal Membrane Oxygenation: a multi-centre study to optimise drug therapy during ECMO

**DOI:** 10.1186/1471-2253-12-29

**Published:** 2012-11-28

**Authors:** Kiran Shekar, Jason A Roberts, Susan Welch, Hergen Buscher, Sam Rudham, Fay Burrows, Sussan Ghassabian, Steven C Wallis, Bianca Levkovich, Vin Pellegrino, Shay McGuinness, Rachael Parke, Eileen Gilder, Adrian G Barnett, James Walsham, Daniel V Mullany, Yoke L Fung, Maree T Smith, John F Fraser

**Affiliations:** 1 Critical Care Research Group, Adult Intensive Care Services, The Prince Charles Hospital and The University of Queensland, Brisbane, Queensland, Australia; 2 Burns Trauma and Critical Care Research Centre, Royal Brisbane and Women’s Hospital and The University of Queensland, Brisbane, Brisbane, Queensland, Australia; 3 Intensive Care Services, St Vincent’s Hospital, Sydney, New South Wales, Australia; 4 Centre for Integrated Preclinical Drug Development, University of Queensland, Brisbane, Queensland, Australia; 5 Intensive Care Services, The Alfred Hospital, Melbourne, Victoria, Australia; 6 Cardiothoracic & Vascular Intensive Care Unit, Auckland City Hospital, Auckland, New Zealand; 7 Institute of Health and Biomedical Innovation, School of Public Health & Social Work, Queensland University of Technology, Brisbane, Queensland, Australia; 8 Intensive Care Services, Princess Alexandra Hospital, Brisbane, Queensland, Australia

**Keywords:** ECMO, Pharmacokinetics, Pharmacodynamics, Antibiotics, Sedatives, Analgesics, Therapeutic failure, Drug toxicity

## Abstract

**Background:**

Given the expanding scope of extracorporeal membrane oxygenation (ECMO) and its variable impact on drug pharmacokinetics as observed in neonatal studies, it is imperative that the effects of the device on the drugs commonly prescribed in the intensive care unit (ICU) are further investigated. Currently, there are no data to confirm the appropriateness of standard drug dosing in adult patients on ECMO. Ineffective drug regimens in these critically ill patients can seriously worsen patient outcomes. This study was designed to describe the pharmacokinetics of the commonly used antibiotic, analgesic and sedative drugs in adult patients receiving ECMO.

**Methods/Design:**

This is a multi-centre, open-label, descriptive pharmacokinetic (PK) study. Eligible patients will be adults treated with ECMO for severe cardiac and/or respiratory failure at five Intensive Care Units in Australia and New Zealand. Patients will receive the study drugs as part of their routine management. Blood samples will be taken from indwelling catheters to investigate plasma concentrations of several antibiotics (ceftriaxone, meropenem, vancomycin, ciprofloxacin, gentamicin, piperacillin-tazobactum, ticarcillin-clavulunate, linezolid, fluconazole, voriconazole, caspofungin, oseltamivir), sedatives and analgesics (midazolam, morphine, fentanyl, propofol, dexmedetomidine, thiopentone). The PK of each drug will be characterised to determine the variability of PK in these patients and to develop dosing guidelines for prescription during ECMO.

**Discussion:**

The evidence-based dosing algorithms generated from this analysis can be evaluated in later clinical studies. This knowledge is vitally important for optimising pharmacotherapy in these most severely ill patients to maximise the opportunity for therapeutic success and minimise the risk of therapeutic failure.

**Trial registration:**

ACTRN12612000559819

## Background

Extracorporeal membrane oxygenation (ECMO) is an invaluable tool for the management of acute severe cardiac and/or respiratory failure in patients failing maximal medical therapy [1]. ECMO is a temporary organ support system and is currently used only for a limited time as a “bridge to recovery”, “bridge to bridge” or “bridge to decision” device [2,3]. Veno-venous (VV) ECMO supports the lungs only, whereas veno-arterial (VA) ECMO provides support for both the heart and lungs. The reported survival for VA ECMO in adult patients with cardiac failure is up to 53% [4]. VV ECMO has a survival rate of up to 71% in patients with severe respiratory failure [5]. The findings of the CESAR trial [6], and the Australian and New Zealand Intensive Care Society ECMO investigator study into H1N1 [5], partly support its role in the advanced management of respiratory failure [7]. With refinements in technology, expanding scope [3] and favourable outcomes in recent studies, it is likely that ECMO may emerge as an indispensible tool in management of critically ill adult patients with cardio-respiratory failure. However, as ECMO finds its niche in adult intensive care as an adjunct to medical therapy, it is important that its effects on the pharmacokinetics (PK) of commonly used intensive care drugs are fully understood to ensure optimal drug therapy and improve patient outcomes. Given that drugs are important for reversing the underlying disease process, unknown interactions between ECMO and pharmacotherapy may seriously impair patient recovery.

In critically ill patients not receiving ECMO, numerous PK studies have demonstrated highly significant changes to drug exposure through interactions between the patient, pathology and the drug [8-11]. The ECMO system introduces additional variables, which are the circuit itself, as well as the systemic inflammation that results from prolonged use of an extracorporeal circuit. Sequestration of drugs in the circuit, increased volume of distribution (Vd) and decreased clearance (CL) are the major PK changes associated with ECMO [12], although the extent of such changes remains poorly characterised. Neonatal studies have reported significant alterations in antibiotic, sedative and analgesic PK [13], but these results may not be generalisable to adult patients due to the developmental and physiologic differences [14].There is emerging data on the altered dose requirements in adult patients on ECMO [15,16]. *In vitro* studies in the neonatal circuit studies highlight the influence that drug properties such as molecular size, degree of ionization at physiological pH, lipophilicity and plasma protein binding have on drug sequestration during ECMO [13,17]. Recently, significant antibiotic, sedative and analgesic drug sequestration has been demonstrated in ECMO circuits used for adult patients [18]. The type and age of circuit components including the type of the pump, oxygenator and tubing, as well as circuit priming, may influence the level of drug sequestration [19-22]. Patient factors such as systemic inflammation, haemodilution, bleeding and transfusion, organ dysfunction and renal replacement therapy (RRT) all add to the clinical challenges of drug dosing during ECMO [23-25].

## Methods/Design

The Antibiotic, Sedative and Analgesic Pharmacokinetics during Extracorporeal Membrane Oxygenation (ASAP ECMO) study is a prospective, multi-centre, open-label, descriptive, PK study of 19 drugs commonly used during ECMO. This study will recruit over 200 patients from 5 ICUs across Australia and New Zealand over 3 years. The primary and secondary aims are:

### Primary aim

• To develop PK models for the antibiotic, sedative and analgesic study drugs and their relevant metabolites described in Table 1 in patients receiving ECMO.

**Table 1:** Study drugs and their relevant metabolites for which population PK models will be developed

**Sedative and analgesics**	**Antiviral/antifungal**	**Antibacterial**
Morphine	Fluconazole	Ceftriaxone
Morphine-3 & 6-glucuronide	Caspofungin	Meropenem
Fentanyl & nor-fentanyl	Voriconazole	Vancomycin
Midazolam	Oseltamivir	Ciprofloxacin
1& 4 hydroxy midazolam	Oseltamivir carboxylate	Gentamicin
Propofol		Piperacillin-tazobactum
Dexmedetomidine		Ticarcillin-clavulunate
Thiopentone		Linezolid

### Secondary aims

• To assess the adequacy of current antibiotic dosing regimens in patients on ECMO.

• To develop guidelines for antibiotic drug dosing during ECMO.

• To develop ECMO-specific sedation protocols.

### Participants

Informed consent will be obtained from study participants or surrogate decision makers as applicable. Eligible patients will be the critically ill admitted to the ICUs at The Prince Charles Hospital, Brisbane, Australia; St Vincent’s Hospital, Sydney, Australia; The Alfred, Melbourne; Auckland City Hospital, Auckland, New Zealand; The Princess Alexandra Hospital, Brisbane, Australia and who have a clinical indication for ECMO. These patients will be receiving sedation and analgesia as part of their routine care, as well as being prescribed a study antibiotic for a clinical indication. A target of 10–12 patients will be enrolled for each study drug. We will examine key antibiotics, analgesics and sedatives (Table 1), and will opportunistically collect blood samples. In some patients, blood samples relating to only antibiotics may be collected, whereas in other patients, samples for analysis of analgesics and sedatives may also be collected.

### Inclusion criteria

• Age > 18 years and < 90 years.

• Currently undergoing ECMO for respiratory and/or cardiac dysfunction.

• Clinical indication for the antibiotics listed in Table 1.

• Clinical indication for the sedatives and analgesics listed in Table 1.

### Exclusion criteria

• No consent

• Known allergy to study drug

• Pregnancy

• Serum bilurubin > 150 μmol/L

• Ongoing massive blood transfusion requirement (> 50% blood volume transfused in the previous 8 hours)

• Therapeutic plasma exchange in the preceding 24 hours

### Drug administration

#### Antibiotics

Antibiotic selection and dosing will be at the discretion of the clinician, based on the clinical context and unit guidelines. Doses will be reconstituted in 10 ml of diluent and given as bolus intravenous (IV) infusion in 50 ml over 30 minutes (except ciprofloxacin, vancomycin and linezolid – 1 or 2 hour IV infusion), or as per local hospital protocols. Antifungals will be infused IV as per local hospital guidelines and oseltamivir will be administered via enteral feeding tube (contents of capsule mixed in 20mL water followed by further 20-50mL water flush).

#### Sedative and analgesic drugs

Sedative and analgesic drugs will be administered according to local policies at each ICU. As a guide, IV infusions and boluses of morphine (10–30 mg/hr) and midazolam (10–30 mg/hr) titrated to a Richmond Agitation Sedation Scale (RASS) [26] of −3 to −4 and a bispectral index (BIS) [27] of 40–45. Patient ventilator interactions may also be used as a guide to titrate sedation especially in patients on venovenous ECMO. Therapeutic paralysis is at the discretion of the treating clinician and will be guided by neuromuscular monitoring.

Additional IV sedation if required may be provided with one of the following agents:

• Propofol IV (10–200 mg/hr)

• Dexmedetomidine IV (1 mcg/kg bolus and 0.1–1.5 mcg/kg/min)

• Fentanyl IV (50–300 mcg/hour) if morphine is discontinued for clinical reasons

• Thiopentone IV (100–200 mg/hour)^*^

^*^ Note- Thiopentone is uncommonly used as an ultimate rescue sedative in some patients on ECMO.

### Sample collection

Blood samples will be drawn from an existing arterial line and collected in 2 ml tubes with a lithium heparin anticoagulant. Where possible, a closed loop Venous Arterial blood Management Protection system (VAMP^TM^, Edward Life sciences, Canada Inc) will be used to minimise blood loss during sampling. The minimum sample volume is 2 mL per time point. Another 0.5 mL of blood will be drawn for each additional drug studied. It is considered unlikely for a patient to be receiving more than 4 study drugs at a given time during PK sampling. Labels for the storage tubes will be provided by the central laboratory and a site specific study number will be allocated to each patient.

#### Blood sampling: antibiotics

**Figure 1:**
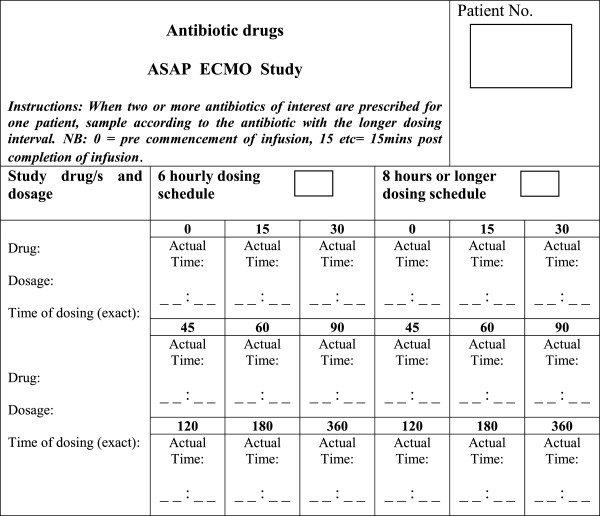
Sampling schedule for antibiotic drugs.

All patients will be sampled over a single dosing period during ECMO. Where two or more antibiotics of interest are prescribed for one patient, data on the timing of administration for both drugs will be collected (Figure 1) with sampling performed according to the antibiotic with the longer dosing interval. For example if vancomycin 1g IV q12h and piperacillin 4.5g IV q6h were both prescribed, sampling would be performed according to the vancomycin 12-hourly dosing schedule.

• Six-hourly dosing schedule – Blood will be sampled from an existing arterial line at the following times: 0, 15, 30, 45, 60, 90, 120, 180 and 360 minutes.

• Eight or 12-hourly dosing schedule – Blood samples will be collected from an existing arterial line at the following times: 0, 15, 30, 45, 60, 90, 120, 180 and 480 minutes.

#### Blood sampling: sedatives and analgesics

**Figure 2:**
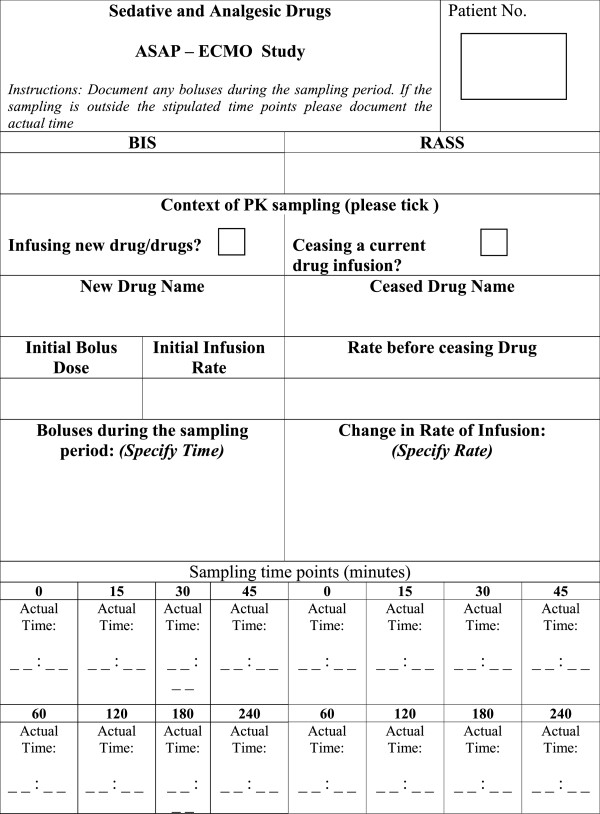
**Sampling schedule for sedative and analgesic drugs.** BIS- bispectral index, RASS- Richmond Agitation Sedation Scale.

Blood samples will be taken from an existing arterial line at 0, 15, 30, 45, 60, 120, 180 and 240 minutes on commencement or cessation of a new sedative drug infusion. The details of drugs, doses and rates of administration to be documented are on the data sheet (Figure 2).

#### Urine specimens

Urinary creatinine clearance collections will be performed for patients not receiving renal replacement therapy as an 8-hour urinary collection. Assay of the urine specimens will be performed by the local pathology service as a surrogate for glomerular filtration rate. For patients receiving RRT the type and dose of the treatment will be documented on the data sheet.

### Data collection and management

**Figure 3:**
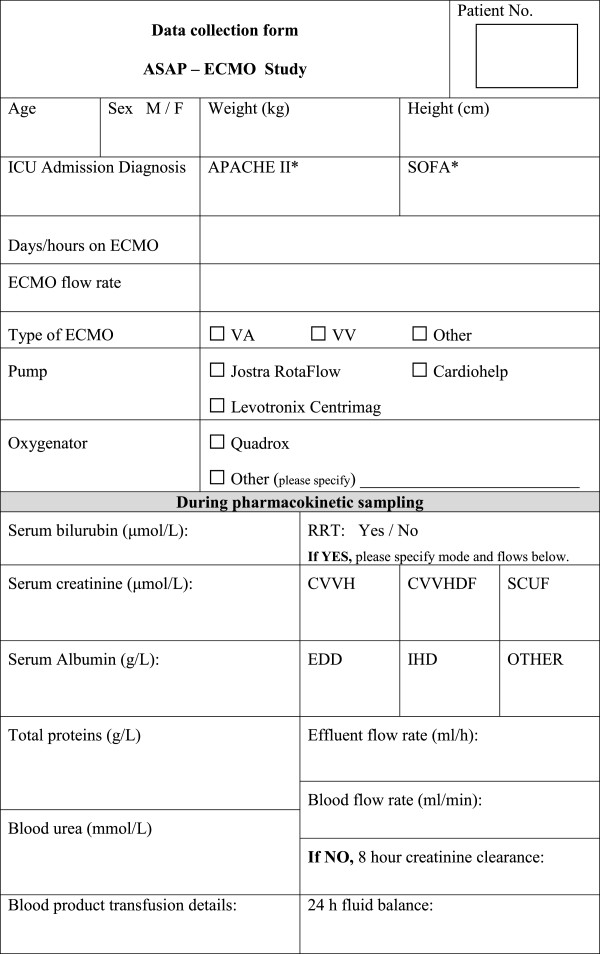
Demographic and clinical data collection form.

For each patient various de-identified clinical and demographic data will be collected by trained research staff at each participating centre and entered onto a data collection form (Figure 3). Each study site will maintain an electronic database for their subjects which will be subsequently consolidated into a single database. The data to be collected includes.

### Demographic data

• Age

• Gender

• Weight

• Height

### Clinical data

• Admission diagnosis, allergies

• Illness severity scores [Sequential Organ Failure Assessment (SOFA)on the day of PK sampling & Acute Physiology and Chronic Health Evaluation (APACHE III) on admission]

• Use of renal replacement therapy

• Sedation scores (RASS, BIS)

### Organ function data

• Serum bilurubin, total protein and albumin concentrations

• Serum creatinine concentrations

• 8-hour urinary creatinine clearance

• 24 h fluid balance and blood product requirements

### ECMO data

• Days of ECMO

• ECMO flows during sampling period

• Type of oxygenator and pump

### Study drug dosing data

• Dose, time and frequency

• Time of sampling

• Day of therapy

### Specimen processing, storage and distribution

Samples will be centrifuged at 3000 g for 10-minutes to separate plasma. Samples containing sedatives and analgesics will be stored locally at −20°C. Samples containing antibiotics will be stored locally at −80 °C. Labels and cryovials will be provided by the central laboratory to the participating sites. Dichlorvas (an inhibitor of plasma esterase activity) will be added to the tubes dedicated to oseltamivir and oseltamivir carboxylate assays. Fluoride oxalate tubes can be used as an alternative.

All samples will then be batched together for transport to the Burns Trauma and Critical Care Research Centre and the Centre for Integrated Preclinical Drug Development at The University of Queensland, Brisbane, Australia. The distribution of samples to the central laboratory will be handled by a commercial clinical trials courier company.

### Pharmacokinetic sample analysis

To reduce the sample burden per patient, validated bioanalytical methods have been developed to quantify multiple drugs and their metabolites selectively and sensitively in small volumes of plasma. A fully automated on-line solid phase extraction (SPE) system (Symbiosis, SPARK Holland) combined with liquid chromatography-mass spectrometry (LC-MS/MS API 5000) to simultaneously quantify morphine, morphine 3-β-D-glucuronide, morphine 6-β-D-glucuronide, midazolam, 1- hydroxymidazolam, 4-hydroxymidazolam, fentanyl and nor-fentanyl in samples of human plasma has been developed [28]. The technique will also be expanded to analyse propofol, thiopentone and dexmedetomidine. Antibiotic concentrations in the collected plasma samples will be determined by separate validated chromatographic assay (HPLC and LC-MS/MS) methods. All samples will be assayed alongside calibration standards and quality control samples, and met the acceptance criteria.

### Statistical and pharmacokinetic analysis

This study aims to explain the variability of PK parameters between patients. Previous experience has shown that 8–12 patients are sufficient for to meet this objective [29]. Plots of drug doses over time for each patient will be used to visually identify trends and outliers. We will perform population PK modelling for each study drug and metabolite of interest using a non-linear mixed effects modelling approach (NONMEM®; Version 6.1, GloboMax LLC, Hanover, MD, USA) as previously described [30-32]. The residuals of the models will be checked to verify model adequacy and influential observations and subjects will be identified. The models will be used to create plots of the predicted mean doses over time. The models will also be used to determine if significant associations exist between the demographic or clinical variables and the pharmacokinetics. Any variables found to have a significant effect on the pharmacokinetics of the drug, will be incorporated into the final pharmacokinetic model. For example, if age is shown to be an important predictor of dose, then we will plot the predicted doses by age group. After developing and testing these PK models, we aim to perform Monte Carlo dosing simulations, which can then form the basis for dosing guidelines for antibiotics and sedative use in patients on ECMO.

### Ethical considerations

Multi-site ethics approval has been obtained (HREC/11/QPCH/121) for the 5 sites in Australia and Lower South Regional Ethics Committee approval for the site in New Zealand (LRS/12/06/020).

### Collaborating organisations

This multi-centre study is co-ordinated by The Critical Care Research Group at The Prince Charles Hospital in Brisbane, Australia. This group will collaborate closely with The Burns Trauma and Critical Care Research Centre, and The Centre for Integrated Preclinical Drug Development, The University of Queensland in Brisbane for antibiotic and sedative drug assays.

### Sample size and power

We estimate that a minimum of 12 patients for each study drug will be sufficient for population PK analysis. A minimum of 12 patients per antibiotic is based on data from previous non-interventional PK studies in critically ill patients [29-31].

## Discussion

This study will identify the sedative and antibiotic drugs whose PK are most influenced by the presence of by ECMO. It will also inform the development of strategies for drug administration using PK and pharmacodynamic principles in critically ill patients receiving ECMO. A lack of understanding of the impact of ECMO on drug Vd and CL may increase the likelihood of therapeutic failure or drug toxicity [33-35]. PK modelling is crucial to drug safety. This study aims to provide the key information for development of evidence-based dosing schedules and sedation protocols for use by clinicians caring for patients receiving ECMO. This study will be complimented by PK studies in the simulated ECMO circuits and ovine models of ECMO [36] which are currently being conducted by the same group. Using the correct sedative agent at an appropriate dose will minimise ICU morbidity, thereby improving patient outcomes [37]. Similarly, the right dose of the right antibiotic [38,39] will not only improve microbiological and clinical cure rates in an individual patient, but may also reduce the emergence of multi-resistant organisms.

## Abbreviations

ECMO: Extracorporeal membrane oxygenation; ICU: Intensive care unit; PK: Pharmacokinetics; Vd: Volume of distribution; CL: Clearance; RASS: Richmond Agitation Sedation Scale; BIS: Bispectral index; SOFA: Sequential Organ Failure Assessment; APACHE: Acute Physiology and Chronic Health Evaluation; HPLC: High performance liquid chromatography; LC-MS/MS: Liquid chromatography tandem mass spectrometry; RRT: Renal replacement therapy.

## Competing interests

The authors declare that they have no competing interests agree.

## Authors’ contributions

KS, JAR, SW, HB, SR designed the study and wrote the initial protocol. SR, BL, VP, SM, FB, RP, EG, AGB, SG, SCW, YLF, JW, DVM, MTS, JFF provided advice and input into the protocol. All authors read and approved the final manuscript.

## Funding

This study is supported in part by funding provided by the Australian and New Zealand College of Anaesthetists, the Intensive Care Foundation, the Prince Charles Hospital Foundation and the Society of Hospital Pharmacists of Australia. Dr Roberts is funded by a Training Research Fellowship from the National Health and Medical Research Council of Australia (569917). Prof Fraser currently holds a Research Fellowship from Queensland Health.

## Pre-publication history

The pre-publication history for this paper can be accessed here:

http://www.biomedcentral.com/1471-2253/12/29/prepub
